# Development of waist circumference percentiles for Japanese children and an examination of their screening utility for childhood metabolic syndrome: a population-based cross-sectional study

**DOI:** 10.1186/s12889-015-2447-1

**Published:** 2015-11-13

**Authors:** Rie Matsushita, Tsuyoshi Isojima, Ryuzo Takaya, Eiichiro Satake, Rie Yamaguchi, Kazuteru Kitsuda, Eiko Nagata, Shinichiro Sano, Toshiki Nakanishi, Yuichi Nakagawa, Takehiko Ohzeki, Tsutomu Ogata, Yasuko Fujisawa

**Affiliations:** Department of Pediatrics, Hamamatsu University School of Medicine, 1-20-1 Handayama, Higashi-ku, Hamamatsu 431-3192 Japan; Department of Pediatrics, Graduate School of Medicine, The University of Tokyo 7-3-1 Hongo, Bunkyo-ku, Tokyo 113-8655 Japan; Department of Pediatrics, Osaka Medical College, 2-7 Daigaku-machi, Takatsuki City, Osaka 569-8686 Japan; Department of Nursing, Kyoritsu Women’s University and Junior College, 3-27 Kanda Jimbocho, Chiyoda-ku, Tokyo 101-0051 Japan

**Keywords:** Childhood metabolic syndrome, Waist circumference, Waist circumference to height ratio, Obesity, Ethnicity

## Abstract

**Background:**

In Japan, waist circumference (WC) percentiles to screen for childhood metabolic syndrome (MetS) are unavailable. The objectives of this study were to develop WC and WC-to-height ratio (WC/Ht) percentile curves by age and sex for Japanese children, and to test their utility in screening for MetS in children with obesity who are otherwise healthy.

**Methods:**

The WC and WC/Ht percentiles were developed using the LMS method of summarizing growth standards, which monitors changing skewness (L), medians (M), and coefficients of variation (S) in childhood distributions. A representative dataset was used, which consisted of 3,634 boys and 3,536 girls aged 4.5–12.75 years in Shizuoka prefecture, Japan, between 2010 and 2012. Children who were obese (355 boys and 230 girls) aged 6–12 years from Osaka prefecture, Japan, were screened for childhood MetS using the new percentiles and the International Diabetes Federation’s (IDF’s) definition of MetS.

**Results:**

The number of participants with certain metabolic abnormalities (high systolic and diastolic blood pressure, and a high level of triglycerides) was significantly higher in boys aged 10–12 years, with a WC ≥ 90th percentile, than among those with a WC < 90th percentile. None of the participants with a WC < 90th percentile exhibited two or more metabolic abnormalities, regardless of their age or sex. Among the participants aged 10–12 years, 11.4 % of boys and 4.4 % of girls with a WC ≥ 90th percentile were diagnosed with MetS.

**Conclusions:**

The new percentiles may have a certain level of potential to screen Japanese children for childhood MetS in accordance with the IDF definition.

**Electronic supplementary material:**

The online version of this article (doi:10.1186/s12889-015-2447-1) contains supplementary material, which is available to authorized users.

## Background

Childhood obesity has been found to be a precursor of health problems in adulthood [1, 2], associated with insulin resistance [3], abnormal glucose metabolism [4], and elevated blood pressure [5]. These abnormalities are identical to the characteristics of adult metabolic syndrome (MetS). Various groups have proposed several criteria of metabolic syndrome in children [6–8] by consensus among experts [9, 10]. Among those, the definition of the International Diabetes Federation (IDF) was most recently published, and has been widely used in research [11–13]; although this definition still has several limitations with this definition [14]. All criteria including the IDF definition, uniformly emphasize the importance of central obesity, defined as an excessive waist circumference (WC). In children and adolescents, percentiles, rather than absolute values of WC by age and sex, are more reliable measures of MetS, and they compensate for large variations throughout development. For example, the IDF defines a WC ≥ 90th percentile across and sex groups as central obesity in children and adolescents. The development of ethnic-specific WC percentiles has been strongly recommended to define central obesity in children across the world [10]. To date, two sets of WC percentiles by age and sex in the Japanese pediatric population have been reported [15, 16]. However, the notable drawback of these percentiles is that the definition of WC as measured at the site of maximum waist narrowing or the top of the iliac crest, which differs from the standard Japanese site, the umbilical position. In Japan the umbilical position is defined as a measurement site of WC for adult MetS criteria in Japan [17]; these criteria are based on the relationship between individuals’ amount of abdominal fat, as measured by computed tomography, and their WC, both at the level of the navel [18]. Although this method is not used internationally [19–21], studies have indicated a strong correlation between the umbilical WC and the risk of MetS in the Japanese population [22, 23]. Based on this background, the umbilical position has been used as the Japanese standard for evaluating WC in health examinations in both adults and children. Given the application of WC percentiles as a screening tool within the health checkup system, the development of a new set of WC percentiles for Japanese children is ideal, using the data collected from measurements at the umbilical position in Japanese children.

This study aimed to develop age- and sex-specific percentiles of WC and WC-to-height ratio (WC/Ht), the latter of which has been reported to be another sensitive indicator of MetS in adulthood and childhood [24–27]. Furthermore, we tested the utility of the developed WC and WC/Ht percentiles to screen for childhood MetS in accordance with the IDF definition.

## Methods

### Development of the WC and WC/Ht percentiles

The Hamamatsu University School of Medicine Ethics Committee approved the study (#22–122). All participants and their parents provided informed consent. Children’s WC measurements were taken as optional assessment during annual health checkups at three randomly selected elementally schools (aged 6–12.75 years), or during 5-year health checkups for children aged 4.5–6 years, which are conducted in two towns in Shizuoka prefecture of Japan, comprising urban, rural, and industrial areas. Each child’s height and weight were measured. At the same time, WC was measured among children who had, and whose parents had agreed to participate in this study through written consent (*N* = 7,418). A total of 229 children were not eligible because written consent was not obtained. The data of children who had foreign citizenships (*N* = 210), in active treatment, or with severe handicaps (*N* = 38) were excluded. Eventually, data on WC percentiles were obtained from 7,170 children (3,634 boys and 3,536 girls).

WC was measured once at the umbilical level to the nearest 0.1 cm, using a constant tension tape by well-trained pediatricians. All participants were measured in the standing position wearing thin school T-shirts. The reason for measuring children wearing clothing was due to the lack of individual privacy during the data collection. In Japan, routine health checkups on school-aged children and adults with wearing thin clothing are regarded within an allowance. Children’s WC/Ht was calculated by dividing WC (cm) by height (cm). The data from seven participants were considered outliers in the dataset using a ± 5 SD cutoff. To obtain smoothed WC percentiles for each gender, we used the LMS method [28], which summarizes the changing distribution by three curves representing skewness (L), the median (M), and the coefficient of variation (S). The method assumes that the data at each age are normally distributed after using the Box–Cox transformation. The values of L, M, and S were constrained to change smoothly with age, and the fitted values were used to construct any required centile curves. Using the penalized likelihood method, three curves (L, M, and S) were fitted as cubic splines by non-linear regression, and the extent of the smoothing was controlled by equivalent degrees of freedom. Fitting and smoothing were done with lmsChartMaker Pro ver. 2.3 (Medical Research Council, London, UK).

### Testing the WC and WC/Ht percentiles

The new WC percentiles were tested on the data set of patients who had visited the pediatric outpatient department of Osaka Medical College Hospital for weight management from 2005 to 2007. The Osama Medical College Ethics Committee approved the study (#1473). Participants consisted of 585 obese children aged 6–12 years of age (355 boys and 230 girls). Obesity was defined as percentage overweight (POW): ≥ 20 % based on the age- and sex-specific standard bodyweight for the height. POW was calculated as 100 × (the measured weight - normal weight)/normal weight (%). Normal weight data based on age-and sex-specific standard body weights for height were obtained from the Ministry of Education, Culture, Sports, Science and Technology. Theoretically, POW that is unique to Japan is not influenced by height, therefore, it is a highly useful index for longitudinal studies and has been widely used in school health checkups to evaluate children’s weight periodically [29]. Height, weight, and WC were recorded. Systolic and diastolic blood pressures (SBP and DBP) were measured in the supine position and the average of three measurements was used for the analysis. After a 12- to 14-hr fast, a blood sample was collected to measure plasma glucose and serum lipid concentrations, namely, high-density lipoprotein (HDL), and triglycerides (TG). Plasma glucose concentrations were determined by the glucose oxidase technique, TG by the enzyme method, and HDL by the selective inhibition method. All laboratory data were evaluated according to the IDF definition of pediatric MetS [9, 10]: 1) a SBP > 130 mmHg or DBP > 85 mmHg; 2), fasting glucose level > 5.6 mM; 3) TG > 1.7 mM; and 4) HDL < 1.03 mM. Data are presented as mean (SD).

### Statistical analysis

The log likelihood ratio test (G test) and the receiver-operating characteristic analysis (ROC) were used to test the validity of the WC and WC/Ht percentiles for screening childhood MetS. JMP for Windows version 8.00 (SAS Institute, Cary, NC) was used for analysis.

## Results

### Reference values for WC and WC/Ht

**Fig. 1:**
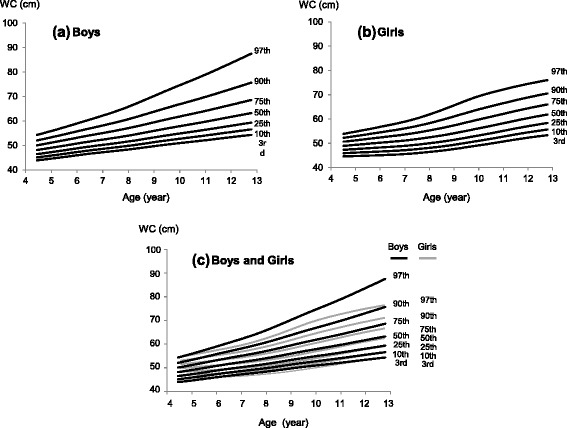
WC percentile curves for Japanese children (aged 4.5–12.75 years). **a** Boys (**b**) Girls (**c**) Boys and Girls

**Fig. 2:**
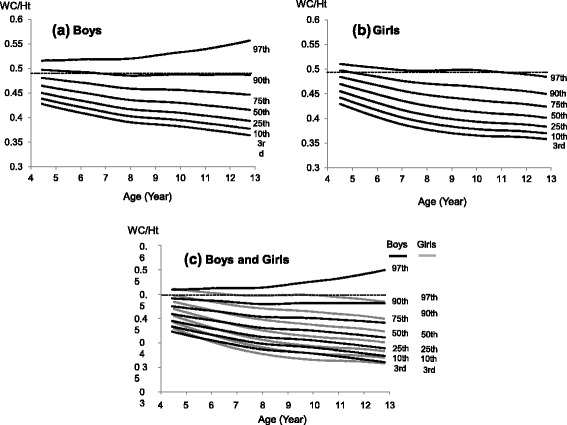
WC/Ht percentile curves for Japanese children (aged 4.5–12.75 years). **a** Boys (**b**) Girls (**c**) Boys and Girls

We found similar distributions of height and weight in our study sample to be similar to the national sample (Table 1) [30]. The LMS parameters for WC and WC/Ht are shown in Additional file 1: Tables S1 and S2, respectively. The WC percentile curves of the boys were greater than those of the girls (Fig. 1). There were no apparent differences between boys and girls in the respective shapes of the curves below the 75th percentile. The shapes of the 90th and 97th percentile curves for boys were linear, whereas those for the girls were sigmoid. All of the WC/Ht percentile curves of boys were greater than were those of girls (Fig. 2). Boys’ WC/Ht 90th percentile curves remained constant at 0.5 for all ages, whereas those for girls declined with age (from 0.5 to 0.45). In contrast, the percentile curves below the 75th percentile for both boys and girls had downward shapes.

**Table 1:** Anthropometric characteristics of the study sample and the Japanese national survey in 2011

Age (years)	Boys					Girls				
	n	Height	Weight	Height	Weight	n	Height	Weight	Height	Weight
				(National data)					(National data)	
4	39	104.8 (3.7)	16.9 (1.6)	ND	ND	23	105.6 (3.5)	17.2 (1.9)	ND	ND
5	61	109.8 (4.2)	18.2 (2.3)	110.5 (4.7)	18.9 (2.6)	59	109.2 (4.6)	18.1 (2.3)	109.5 (4.7)	18.5 (2.5)
6	266	115.9 (4.5)	20.8 (3.0)	116.6 (4.9)	21.3 (3.3)	270	115.5 (4.3)	20.4 (2.5)	115.6 (4.9)	20.8 (3.1)
7	571	121.3 (5.1)	23.3 (3.5)	122.6 (5.3)	24.0 (4.2)	559	120.2 (4.6)	22.2 (2.9)	121.6 (5.1)	23.4 (3.8)
8	628	127.1 (5.1)	26.3 (4.6)	128.2 (5.4)	27.0 (5.0)	624	126.2 (5.1)	25.2 (3.8)	127.4 (5.5)	26.4 (4.7)
9	635	132.3 (5.4)	29.5 (5.7)	133.5 (5.7)	30.3 (6.0)	606	132.4 (6.1)	28.8 (5.1)	133.5 (6.2)	29.8 (5.8)
10	610	137.4 (6.1)	32.7 (6.4)	138.8 (6.1)	33.8 (7.3)	588	139.3 (6.7)	32.9 (6.3)	140.2 (6.8)	34.0 (6.9)
11	602	143.7 (6.7)	37.4 (8.1)	145.0 (7.1)	38.0 (8.2)	583	145.5 (6.6)	37.2 (7.0)	146.7 (6.7)	38.8 (7.8)
12	222	149.4 (7.8)	41.7 (9.8)	152.3 (8.0)	43.8 (9.7)	224	149.8 (5.9)	40.4 (7.0)	151.9 (5.9)	43.6 (8.1)

*n* Number of participants, *ND* No data, values are presented as means (SD)

Participants who were obese (355 boys and 230 girls) were separated by sex and then divided into two age groups—aged 6–9 years and 10–12 years—according to the age-grouping in the IDF definition of childhood MetS [9, 10]. Each group was further divided into two groups using the new WC percentiles (below and above the 90th percentiles), namely, the IDF’s cutoff, to determine their utility as a screening tool for MetS. The number of metabolic risk factors in participants with a WC ≥ 90th percentile was higher, regardless of their age, in comparison with that of risk factors among children with a WC < 90th percentile (Table 2). Among children aged 10–12 years with a WC ≥ 90th percentile, 16 (11.4 %) of the 140 boys were diagnosed with MetS. There were no boys with a WC < 90th percentile, with two or more metabolic risk factors (*p* < 0.05). Three (4.4 %) of the 68 girls with a WC ≥ 90th percentile exhibited two or more metabolic risk factors that approached a MetS diagnosis whereas none of the girls with a WC < 90th percentile had two or more metabolic risk factors, but the difference was not statistically significant. In the group aged 6–9 years with a xWC ≥ 90th percentile, 7 (3.9 %) of the 180 boys and 2 (1.3 %) of the 154 girls exhibited two or more risk factors. Then, validity was tested using the integrated data obtained from all the participants. The number of participants with a cluster (two or more) of metabolic abnormality was higher among the group with a WC ≥ 90th percentile than that among those with a WC < 90th percentile (*p* = 0.0362).

**Table 2:** Clinical characteristics of the participants by the WC 90th percentile

Age Group		Boys				Girls			
		WC percentile				WC percentile			
		< 90th		90-100th		< 90th		90-100th	
6-9 yr	n	14		180		2		154	
	Systolic BP < 130 mmHg	0/14	(0.0)	12/180	(6.7)	0/2	(0.0)	9/154	(5.8)
	Dyastolic BP < 85 mmHg	1/14	(7.1)	9/180	(5.0)	0/2	(0.0)	12/154	(7.8)
	BS < 5.6 mM	2/14	(14.3)	36/180	(20.0)	0/2	(0.0)	17/154	(11.0)
	TG < 1.7 mM	0/14	(0.0)	10/180	(5.6)	0/2	(0.0)	5/154	(3.3)
	HDL-C <1.03 mM	0/14	(0.0)	4/180	(2.2)	0/2	(0.0)	5/154	(3.3)
	Two or more metabolic risk factors	0/14	(0.0)	7/180	(3.9)	0/2	(0.0)	2/154	(1.3)
10-12 yr	n	21		140		6		68	
	Systolic BP < 130 mmHg	0/21	(0.0)	26/140	(18.6)	0/6	(0.0)	12/68	(17.7)
	Dyastolic BP < 85 mmHg	0/21	(0.0)	20/140	(14.3)	0/6	(0.0)	5/68	(7.4)
	BS < 5.6 mM	6/21	(28.6)	31/140	(22.1)	1/6	(16.7)	14/68	(20.6)
	TG < 1.7 mM	0/21	(0.0)	15/140	(10.7)	0/6	(0.0)	5/68	(7.4)
	HDL-C <1.03 mM	0/21	(0.0)	6/140	(4.3)	0/6	(0.0)	1/68	(1.5)
	Two or more metabolic risk factors	0/21	(0.0)	16/140	(11.4)	0/6	(0.0)	3/68	(4.4)

The numbers in the parentheses show the percentages of children

*n* Number of participants, *WC* Waist circumference, *BP* Blood pressure, *BS* Blood sugar, *TG* Triglyceride, *HDL-C* High-density lipoprotein cholesterol

Next, we tested the use of the WC 97th percentile cutoff (Table 3) because a 90th percentile cutoff for WC might lead to an over-diagnosis of central obesity with application on Japanese children, whose WC is relatively smaller than those of children in western countries. Metabolic abnormalities were present in both WC < 97th and WC ≥ 97th percentile groups of boys aged 6–9 and 10–12 years, and of girls aged 10–12 years, unlike in the case of the WC 90th percentile cutoff. Two (3.4 %) of the 59 boys (aged 6–9 years) and 7 (6.9 %) of the 101 boys (aged 10–12 years) with a WC < 97th percentile cutoff exhibited a cluster of metabolic risk factors. None of the girls aged 6–9 years presented with two or more metabolic risk factors, but 2 (7.7 %) of the 26 girls aged 10–12 years did so, even when their WC was below the cutoff. In accordance with these results, the validity test on a WC < 97th percentile cutoff failed to show a statistical significance (*p* = 0.689).

**Table 3:** Clinical characteristics of the participants by the WC 97th percentile

Age Group		Boys				Girls			
		WC percentile				WC percentile			
		< 97th		97-100th		< 97th		97-100th	
6-9 yr	n	59		135		23		133	
	Systolic BP < 130 mmHg	2/59	(3.4)	10/135	(7.4)	1/23	(4.4)	8/133	(6.0)
	Dyastolic BP < 85 mmHg	5/59	(8.5)	5/135	(3.7)	2/23	(8.7)	10/133	(7.5)
	BS < 5.6 mM	17/59	(28.8)	21/135	(15.6)	1/23	(4.4)	16/133	(12.0)
	TG < 1.7 mM	1/59	(1.7)	9/135	(6.7)	0/23	(0.0)	5/133	(3.8)
	HDL-C <1.03 mM	0/59	(0.0)	4/135	(3.0)	0/23	(0.0)	5/133	(3.8)
	Two or more metabolic risk factors	2/59	(3.4)	5/135	(3.7)	0/23	(0.0)	2/133	(1.5)
10-12 yr	n	101		60		26		48	
	Systolic BP < 130 mmHg	15/101	(14.9)	11/60	(18.3)	4/26	(15.4)	8/48	(16.7)
	Dyastolic BP < 85 mmHg	10/101	(9.9)	10/60	(16.7)	1/26	(3.9)	4/48	(8.3)
	BS < 5.6 mM	20/101	(19.8)	17/60	(28.3)	5/26 (19.2)	10/48	(20.8)	
	TG < 1.7 mM	5/101	(5.0)	10/60	(16.7)	2/26	(7.7)	3/48	(6.3)
	HDL-C <1.03 mM	3/101	(3.0)	3/60	(5.0)	1/26	(3.9)	0/48	(0.0)
	Two or more metabolic risk factors	7/101	(6.9)	9/60	(15.0)	2/26	(7.7)	1/48	(2.1)

The numbers in the parentheses show the percentages of children

*n* number of participants, *WC* Waist circumference, *BP* Blood pressure, *BS* Blood sugar, *TG* Triglyceride, *HDL-C* High-density lipoprotein cholesterol

We also tested the validity of the WC/Ht ratio in screening for MetS in obese participants, using the 90th percentile cutoff (Table 4) and 97th percentile cutoff (Table 5). In the group aged 10–12 years, 16 (10.7 %) of the 149 boys and 3 (4.1 %) of the 73 girls exhibited two or more metabolic risk factors, as did 7 (3.9 %) of the 180 boys and 2 (1.3 %) of the 155 girls aged 6–9 years with a WC/Ht ≥ 90th percentile. The percentage of participants with two or more metabolic risk factors over the cutoff (WC/Ht: 90th percentile) was similar to that obtained using the WC 90th percentile: None of the participants with a WC/Ht < 90th percentile, regardless of age, exhibited two or more metabolic abnormalities: however no statistical significance was obtained for the validity test (*p* = 0.0993). When using WC/Ht 97th percentile as the cutoff, metabolic abnormalities were present in children aged 10–12 years in the group with above or below cutoff. Four of the 78 boys and 2 of the 15 girls, even with WC below the cutoff point exhibited two or more metabolic risk factors. Predictably, no statistical significance was obtained for the validity test (*p* = 0.6381).

**Table 4:** Clinical characteristics of the participants by the WC/Ht 90th percentile

Age Group		Boys				Girls			
		WC/Ht percentile				WC/Ht percentile			
		< 90th		90-100th		< 90th		90-100th	
6-9 yr n		14		180		1		155	
	Systolic BP < 130 mmHg	0/14	(0.0)	12/180	(6.7)	0/1	(0.0)	9/155	(5.8)
	Dyastolic BP < 85 mmHg	1/14	(7.1)	9/180	(5.0)	0/1	(0.0)	12/155	(7.7)
	BS < 5.6 mM	2/14	(14.3)	36/180	(20.0)	0/1	(0.0)	17/155	(11.0)
	TG < 1.7 mM	0/14	(0.0)	10/180	(5.6)	0/1	(0.0)	5/155	(3.2)
	HDL-C <1.03 mM	0/14	(0.0)	4/180	(2.2)	0/1	(0.0)	5/155	(3.2)
	Two or more metabolic risk factors	0/14	(0.0)	7/180	(3.9)	0/1	(0.0)	2/155	(1.3)
10-12 yr n		12		149		1		73	
	Systolic BP < 130 mmHg	0/12	(0.0)	26/149	(17.5)	0/1	(0.0)	12/73	(16.4)
	Dyastolic BP < 85 mmHg	0/12	(0.0)	20/149	(13.4)	0/1	(0.0)	5/73	(6.9)
	BS < 5.6 mM	3/12	(25.0)	34/149	(22.8)	0/1	(0.0)	15/73	(20.6)
	TG < 1.7 mM	0/12	(0.0)	15/149	(10.1)	0/1	(0.0)	5/73	(6.9)
	HDL-C <1.03 mM	0/12	(0.0)	6/149	(4.0)	0/1	(0.0)	1/73	(1.8)
	Two or more metabolic risk factors	0/12	(0.0)	16/149	(10.7)	0/1	(0.0)	3/73	(4.1)

The numbers in the parentheses show the percentages of children

*WC* Waist circumference, *BP* Blood pressure, *BS* Blood sugar, *TG* Triglyceride, *HDL-C* High-density lipoprotein cholesterol, *ns* Not significant

**Table 5:** Clinical characteristics of the participants by the WC/Ht 97th percentile

Age Group		Boys				Girls			
		WC/Ht percentile				WC/Ht percentile			
		< 97th		97-100th		< 97th		97-100th	
6-9 yr	n	40		154		14		142	
	Systolic BP < 130 mmHg	2/40	(5.0)	10/154	(6.5)	0/1	(0.0)	9/142	(6.3)
	Dyastolic BP < 85 mmHg	2/40	(5.0)	8/154	(5.2)	1/14	(7.1)	11/142	(7.8)
	BS < 5.6 mM	7/40	(17.5)	31/154	(20.1)	0/14	(0.0)	17/142	(12.0)
	TG < 1.7 mM	0/40	(0.0)	10/154	(6.5)	0/14	(0.0)	5/142	(3.5)
	HDL-C <1.03 mM	0/40	(0.0)	4/154	(2.6)	0/14	(0.0)	5/142	(3.5)
	Two or more metabolic risk factors	0/40	(0.0)	7/154	(4.6)	0/14	(0.0)	2/142	(1.4)
10-12 yr	n	78		83		38		78	
	Systolic BP < 130 mmHg	11/78	(14.1)	15/83	(18.1)	2/15	(13.3)	10/59	(17.0)
	Dyastolic BP < 85 mmHg	6/78	(7.7)	14/83	(16.9)	0/15	(0.0)	5/59	(8.5)
	BS < 5.6 mM	17/78	(21.8)	20/83	(24.1)	4/15	(26.7)	11/59	(18.6)
	TG < 1.7 mM	3/78	(3.9)	12/83	(14.5)	2/15	(13.3)	3/59	(5.1)
	HDL-C <1.03 mM	0/78	(0.0)	6/83	(7.2)	1/15	(6.7)	0/59	(0.0)
	Two or more metabolic risk factors	4/78	(5.1)	12/83	(14.5)	2/15	(13.3)	1/59	(1.7)

The numbers in the parentheses show the percentages of children

*n* number of participants, *WC* Waist circumference, *BP* Blood pressure, *BS* Blood sugar, *TG* Triglyceride, *HDL-C* High-density lipoprotein cholesterol

Furthermore, we performed ROC analysis to support that the notion that WC at the 90th percentile was the most appropriate value for screening childhood MetS [explanatory variable: higher or lower than the cutoff WC percentile (75, 90, or 97th), and supervised variable: two or more metabolic abnormalities, using combined data from boys and girls, regardless of age]. The confidence interval for the area under the ROC the curve (AUC) was 0.505 (95 % CI: 0.501–0.510, sensitivity; 1.000, specificity; 0.011) for WC 75th percentile cutoff, 0.539 (95 % CI: 0.528–0.550, sensitivity; 1.000, specificity; 0.077) for WC 90th percentile cutoff, or 0.482 (95 % CI: 0.387–0.576, sensitivity; 0.607, specificity; 0.356) for WC 95th percentile cutoff. Taken together, among those cutoff points, WC 90th percentile cutoff may have a certain level of potential for screening childhood MetS.

## Discussion

This was the first trial to develop WC percentiles by age and sex in the Japanese pediatric population using a new dataset of measurements at the standardized Japanese site (the umbilical site). We showed that the new WC percentiles have the potential to distinguish children with MetS from obese children who are otherwise healthy according to the IDF criteria, which are used worldwide.

Currently, there is no consensus on the WC measurement site for the pediatric population, although several positions are used [31]. In response to this situation, the international definitions of childhood MetS, such as the IDF criteria, do not define a measurement position for WC. Furthermore, the relationship between WC values and cardiometabolic risk factors do not appear to be affected by measurement site, including at the narrowest part of the waist, the midpoint between the lowest rib and iliac crest, or just above the iliac crest and the umbilical level [32]. In Japan, the adult criteria for MetS included a WC measurement at the umbilical site for the diagnosis [17], based on a previous study [18]; therefore, the umbilical position is used as the standard position for all ages at health examinations. To date, two sets of WC percentiles by age and sex in the Japanese pediatric population have been reported [15, 16]; however, those studies used WC measurements from the narrowest part of the waist or the top of the iliac crest, not at the umbilical site. Therefore, it should be emphasized that the new WC percentiles are more useful for screening childhood MetS through school health checkups.

Sex differences in visceral fat as well as WC are believed to appear at puberty [33–37], with boys showing more visceral fat than do girls, due to sex differences in hormonal concentrations [38]; however, in prepubescent children, visceral fat levels are roughly similar. It is noteworthy that the actual value of the boys’ WC at the 90th percentile in this study becomes greater than that of girls at around the age of six (Fig. 1 and Additional file 1); nevertheless, gender differences in sex hormones are thought to be negligible at this age. A study of WC percentiles in Japanese children reported similar results [16], although the WC measurements differed from those in this study. Turkish boys also showed a predominance of central adiposity throughout early childhood [39], indicating the presence of sex differences in visceral fat deposits before puberty, which is consistent with previous findings [40]. This study underscores the importance of developing sex-specific WC percentiles by age from early childhood to puberty. It is worth mentioning that pubertal status can affect metabolic parameters, including blood pressure, lipid profile, and glucose metabolism [41, 42]. In this study, the pubertal status of the participants was not considered. One reason is that pubertal status is not included in the IDF definition of MetS. Besides, because of the lack of individual privacy during the school health checkups, puberty status could not be assessed. Further studies on the screening of childhood MetS, so as to examine the strength of the effect of pubertal status on metabolic parameters are necessary.

The 90th percentile for WC has been reported as a reliable cutoff for screening metabolic abnormalities in children [43–45]. Because there were no MetS subjects in boys and girls with a WC < 90th percentile, we demonstrated that the cutoff point of WC at the 90th percentile involved a certain level of reliability for distinguishing children with MetS, according to the IDF criteria, from obese children who were otherwise health, When the WC cutoff was set at the 97th percentile, some of the participants below that percentile were found to have multiple metabolic abnormalities. This finding indicated that the WC at the 97th percentile was inadequate in distinguishing children with MetS from those who were obese but were otherwise healthy. The validity of WC at the 90th percentile was supported by the results obtained from the statistical analysis, although data from all the participants were integrated, due to extremely small number of non-obese subjects. These results are compatible with previous reports that a slight increase in metabolic risk factors begins at the 75th WC percentile, while a considerable increase begins at the 90th percentile [46, 47]. Further studies are needed to clarify specific cutoffs by ethnicity, while satisfying sensitivity and specificity criteria to screen for childhood MetS.

The WC/Ht ratio has the potential to be a better screening index for metabolic risks in adults than does WC [27]. Since WC/Ht does not change dramatically in accordance with the ages of children and adolescents, specific references are unnecessary, giving it an advantage in screening for childhood MetS. The WC/Ht cutoff was 0.5, which was created based on adult studies showing an association between WC/Ht and metabolic risk [25, 26], and the same 0.5 cutoff can be used across all ages in pediatric populations [27, 48]. In our study, the WC/Ht 90th percentile of the boys was constant at 0.5 across age. Therefore, it can be used as a simple screening parameter to predict a cluster of metabolic risk factors in boys. The 90th percentile curves of the girls in our study were not constant at 0.5 across age, indicating that the value of 0.5 WC/Ht was not a good screening tool for MetS in girls. This result is consistent with a study showing that the WC/Ht cutoff was not constant at 0.5 for all ages in [49] suggesting the need for specific age and sex references.

There are some limitations in the present study. The first limitation is that the new percentile curves were based on a sample from only one prefecture in Japan, not from randomly selected regions across the entire country, which restricts their generalizability as national references. Therefore, it might be difficult to apply the results of this study to Japanese children in the general population. However, mean heights and weights were similar to those of Japanese children of the same age in the general population. Furthermore, several studies reported that the distributions of height and weight in school-aged children of Shizuoka prefecture were similar to those in the national survey [50, 51]. Therefore, the new WC percentiles from this study have the potential to be of great value in their application by clinicians to the general population of Japanese children. Studies that use data collected from several other regions of Japan are needed to establish national references. The second limitation of the present study is that the samples used for the utility evaluation of the new WC percentiles were obtained from only one site, Osaka prefecture, and did not include participants who were not overweight, resulting in a relatively small number of children being classified below the WC cutoff values (90th or 97th percentiles). Therefore, we cannot provide firm evidence supporting the use of the new set of WC percentiles to screen for childhood MetS (in accordance with the IDF definition) throughout the country. It should also be taken into account that the validity of the WC and WC/Ht percentiles is retrospective (using the pooled data collected before the establishment of those percentiles). Considering that the incidence of childhood obesity in Japan has either remained the same or decreased since 2000 [52], this trial of validity should be acceptable. It must be mentioned that the result of poor AUC values and low specificity caused by relatively small number of MetS subjects is a significant weakness in this study. Given that the WC 90th percentile cutoff showed a good sensitivity (1.00) with relatively high AUC values among three cutoff points (75th, 90th, or 97th), it might be possible that this cutoff values has a potential for the screening of childhood MetS. Future studies using a data set of newly recruited subjects, including non-obese subjects from the entire country are necessary to ensure the reliability of the WC 90th percentile cutoff for screening childhood MetS in Japan.

## Conclusion

In summary, this study provides a new set of pediatric WC and WC/Ht percentiles specific to age and sex, based on the data collected from Japanese children aged 4.5–12.75 years. Children’s measurements of their WC were taken at the umbilical site, which is the site used universally in Japan. The WC percentile was effective in distinguishing participants with childhood MetS from children who were obese but otherwise healthy, based on the IDF criteria that are used worldwide.

## Additional file

Additional file 1:
**Table S1.** Box–Cox values for power (L), adjusted mean (M), and coefficient of variation (S) of WC for the Japanese children of boys and girls from 4.50 to 12.75 years of age. **Table S2.** Box–Cox values for power (L), adjusted mean (M), and coefficient of variation (S) of WC/Ht for the Japanese children of boys and girls from 4.50 to 12.75 years of age. (DOCX 179 kb)

## Abbreviations

LMS: Skewness (L), medians (M), and coefficients of variation (S) of growth distributions, referred to in the LMS method
MetS: Metabolic syndrome
WC: Waist circumference
WC/Ht: Waist circumference to height ratio

## References

[1] Dietz WH (1998). Health consequences of obesity in youth: childhood predictors of adult disease. Pediatrics.

[2] Must A, Strauss RS (1999). Risks and consequences of childhood and adolescent obesity. Int J Obes Relat Metab Disord.

[3] Sinaiko AR, Caprio S (2012). Insulin resistance. J Pediatr.

[4] Sinha R, Fisch G, Teague B, Tamborlane WV, Banyas B, Allen K (2002). Prevalence of impaired glucose tolerance among children and adolescents with marked obesity. N Engl J Med.

[5] Sorof JM, Lai D, Turner J, Poffenbarger T, Portman RJ (2004). Overweight, ethnicity, and the prevalence of hypertension in school-aged children. Pediatrics.

[6] Defining the problem (2000). Obesity: preventing and managing the global epidemic.

[7] de Ferranti SD, Gauvreau K, Ludwig DS, Neufeld EJ, Newburger JW, Rifai N (2004). Prevalence of the metabolic syndrome in American adolescents: findings from the Third National Health and Nutrition Examination Survey. Circulation.

[8] Weiss R, Dziura J, Burgert TS, Tamborlane WV, Taksali SE, Yeckel CW (2004). Obesity and the metabolic syndrome in children and adolescents. N Engl J Med.

[9] Zimmet P, Alberti G, Kaufman F, Tajima N, Silink M, Arslanian S (2007). The metabolic syndrome in children and adolescents. Lancet.

[10] Zimmet P, Alberti KG, Kaufman F, Tajima N, Silink M, Arslanian S (2007). The metabolic syndrome in children and adolescents - an IDF consensus report. Pediatr Diabetes.

[11] Kurtoglu S, Akin L, Kendirci M, Hatipoglu N, Elmali F, Mazicioglu M (2012). The absence of insulin resistance in metabolic syndrome definition leads to underdiagnosing of metabolic risk in obese patients. Eur J Pediatr.

[12] Sangun Ö, Dündar B, Kösker M, Pirgon Ö, Dündar N (2011). Prevalence of metabolic syndrome in obese children and adolescents using three different criteria and evaluation of risk factors. J Clin Res Pediatr Endocrinol.

[13] Ford ES, Li C, Zhao G, Pearson WS, Mokdad AH (2008). Prevalence of the metabolic syndrome among U.S. adolescents using the definition from the International Diabetes Federation. Diabetes Care.

[14] Weiss R, Bremer AA, Lustig RH (2013). What is metabolic syndrome, and why are children getting it?. Ann N Y Acad Sci.

[15] Anzo M, Inokuchi M, Matsuo N, Takayama JI, Hasegawa T (2014). Waist circumference centiles by age and sex for Japanese children based on the 1978–1981 cross-sectional national survey data. Ann Hum Biol.

[16] Inokuchi M, Matsuo N, Anzo M, Takayama JI, Hasegawa T (2007). Age-dependent percentile for waist circumference for Japanese children based on the 1992–1994 cross-sectional national survey data. Eur J Pediatr.

[17] Matsuzawa Y (2005). Metabolic syndrome-definition and diagnostic criteria in Japan. J Atheroscler Thromb.

[18] Nagaretani H, Nakamura T, Funahashi T, Kotani K, Miyanaga M, Tokunaga K (2001). Visceral fat is a major contributor for multiple risk factor clustering in Japanese men with impaired glucose tolerance. Diabetes Care.

[19] Matsushita Y, Tomita K, Yokoyama T, Mizoue T (2010). Relations between waist circumference at four sites and metabolic risk factors. Obesity (Silver Spring).

[20] Okazawa T, Iwata M, Matsushita Y, Kamura Y, Kato H, Okazawa S (2013). Aging attenuates the association of central obesity with the accumulation of metabolic risk factors when assessed using the waist circumference measured at the umbilical level (the Japanese standard method). Nutr Diabetes.

[21] Yokoyama H, Hirose H, Kanda T, Kawabe H, Saito I (2011). Relationship between waist circumferences measured at the umbilical level and midway between the ribs and iliac crest - a solution to the debate on optimal waist circumference standards in the diagnostic criteria of metabolic syndrome in Japan. J Atheroscler Thromb.

[22] Eguchi Y, Eguchi T, Mizuta T, Ide Y, Yasutake T, Iwakiri R (2006). Visceral fat accumulation and insulin resistance are important factors in nonalcoholic fatty liver disease. J Gastroenterol.

[23] Ninomiya T, Kubo M, Doi Y, Yonemoto K, Tanizaki Y, Rahman M (2007). Impact of metabolic syndrome on the development of cardiovascular disease in a general Japanese population: the Hisayama study. Stroke.

[24] Hara M, Saitou E, Iwata F, Okada T, Harada K (2002). Waist-to-height ratio is the best predictor of cardiovascular disease risk factors in Japanese schoolchildren. J Atheroscler Thromb.

[25] Hsieh SD, Yoshinaga H, Muto T (2003). Waist-to-height ratio, a simple and practical index for assessing central fat distribution and metabolic risk in Japanese men and women. Int J Obes Relat Metab Disord.

[26] Hsieh SD, Yoshinaga H (1995). Waist/height ratio as a simple and useful predictor of coronary heart disease risk factors in women. Intern Med.

[27] Ashwell M, Gunn P, Gibson S (2012). Waist-to-height ratio is a better screening tool than waist circumference and BMI for adult cardiometabolic risk factors: systematic review and meta-analysis. Obes Rev.

[28] Cole TJ (1990). The LMS, method for constructing normalized growth standards. Eur J Clin Nutr.

[29] Asayama K, Ozeki T, Sugihara S, Ito K, Okada T, Tamai H (2003). Criteria for medical intervention in obese children: a new definition of ‘obesity disease’ in Japanese children. Peadiatr Int.

[30] Annual Report of School Health Statistics Research . 2012. http://www.e-stat.go.jp/SG1/estat/List.

[31] Johnson ST, Kuk JL, Mackenzie KA, Huang TT, Rosychuk RJ, Ball GD (2010). Metabolic risk varies according to waist circumference measurement site in overweight boys and girls. J Pediatr.

[32] Harrington DM, Staiano AE, Broyles ST, Gupta AK, Katzmarzyk PT (2013). Waist circumference measurement site does not affect relationships with visceral adiposity and cardiometabolic risk factors in children. Pediatr Obes.

[33] Goran MI (1999). Visceral fat in prepubertal children: influence of obesity, anthropometry, ethnicity, gender, diet, and growth. Am J Hum Biol.

[34] Goran MI, Gower BA (1999). Relation between visceral fat and disease risk in children and adolescents. Am J Clin Nutr.

[35] Satake E, Nakagawa Y, Kubota A, Saegusa H, Sano S, Ohzeki T (2010). Age and sex differences in fat distribution in non-obese Japanese children. J Pediatr Endocrinol Metab.

[36] Staiano AE, Katzmarzyk PT (2012). Ethnic and sex differences in body fat and visceral and subcutaneous adiposity in children and adolescents. Int J Obes (Lond).

[37] Moreno LA, Fleta J, Mur L, Rodríquez G, Sarría A, Bueno M (1999). Waist circumference values in Spanish children--gender related differences. Eur J Clin Nutr.

[38] Roemmich JN, Rogol AD (1999). Hormonal changes during puberty and their relationship to fat distribution. Am J Hum Biol.

[39] Hatipoglu N, Mazicioglu MM, Poyrazoglu S, Borlu A, Horoz D, Kurtoglu S (2013). Waist circumference percentiles among Turkish children under the age of 6 years. Eur J Pediatr.

[40] Taylor RW, Gold E, Manning P, Goulding A (1997). Gender differences in body fat content are present well before puberty. Int J Obes Relat Metab Disord.

[41] Reinehr T, Toschke AM (2009). Onset of puberty and cardiovascular risk factors in untreated obese children and adolescents: a 1-year follow-up study. Arch Pediatr Adolesc Med.

[42] Reinehr T, Wolters B, Knop C, Lass N, Holl RW (2015). Strong effect of pubertal status on metabolic health in obese children: a longitudinal study. J Clin Endocrinol Metab.

[43] Bassali R, Waller JL, Gower B, Allison J, Davis CL (2010). Utility of waist circumference percentile for risk evaluation in obese children. Int J Pediatr Obes.

[44] Burns SF, Arslanian SA (2009). Waist circumference, atherogenic lipoproteins, and vascular smooth muscle biomarkers in children. J Clin Endocrinol Metab.

[45] Zhang YX, Wang SR (2014). Comparison of blood pressure levels among children and adolescents with different body mass index and waist circumference: study in a large sample in Shandong. China Eur J Nutr.

[46] Ma GS, Ji CY, Ma J, Mi J, Yt Sung R, Xiong F (2010). Waist circumference reference values for screening cardiovascular risk factors in Chinese children and adolescents. Biomed Environ Sci.

[47] Liu A, Hills AP, Hu X, Li Y, Du L, Xu Y (2010). Waist circumference cut-off values for the prediction of cardiovascular risk factors clustering in Chinese school-aged children: a cross-sectional study. BMC Public Health.

[48] McCarthy HD, Ashwell M (2006). A study of central fatness using waist-to-height ratios in UK children and adolescents over two decades supports the simple message—‘keep your waist circumference to less than half your height.’. Int J Obes (Lond).

[49] Li C, Ford ES, Mokdad AH, Cook S (2006). Recent trends in waist circumference and waist-height ratio among US children and adolescents. Pediatrics.

[50] Kouda K, Nakamura H, Nishio N, Fujita Y, Takeuchi H, Iki M (2010). Trends in body mass index, blood pressure, and serum lipids in Japanese children: Iwata population-based annual screening (1993–2008). J Epidemiol.

[51] Kouda K, Nakamura H, Fujita Y, Iki M (2012). Relationship between body mass index at age 3 years and body composition at age 11 years among Japanese children: the Shizuoka population-based study. J Epidemiol.

[52] Yoshinaga M, Ichiki T, Tanaka Y, Hazeki D, Horigome H, Takahashi H (2010). Prevalence of childhood obesity from 1978 to 2007 in Japan. Pediatr Int.

