# Supplementary material for: Development of waist circumference percentiles for Japanese children and an examination of their screening utility for childhood metabolic syndrome: a population-based cross-sectional study
Source: BMC Public Health. 2015 Nov 13;15:1121. doi: 10.1186/s12889-015-2447-1 (PMC4644326; doi:10.1186/s12889-015-2447-1)
Supplement: Supplementary file 1 [file 12889_2015_2447_MOESM1_ESM.docx]

Additional file 1: Box–Cox values for power (L), adjusted mean (M), and coefficient of variation (S) of WC for the Japanese children of boys and girls from 4.50 to 12.75 years of age.

Age (years)	Boys			Girls		
	L	M	S	L	M	S
4.50	-2.61	48.21	0.055	-0.36	48.94	0.050
4.75	-2.73	48.62	0.057	-0.47	49.15	0.052
5.00	-2.86	49.04	0.058	-0.59	49.37	0.054
5.25	-2.98	49.46	0.059	-0.70	49.59	0.056
5.50	-3.10	49.89	0.06	-0.82	49.81	0.057
5.75	-3.22	50.32	0.062	-0.93	50.03	0.059
6.00	-3.34	50.76	0.063	-1.05	50.25	0.061
6.25	-3.44	51.19	0.064	-1.17	50.46	0.063
6.50	-3.54	51.60	0.065	-1.30	50.67	0.064
6.75	-3.62	51.99	0.066	-1.43	50.90	0.066
7.00	-3.70	52.38	0.068	-1.57	51.15	0.067
7.25	-3.75	52.76	0.069	-1.72	51.43	0.069
7.50	-3.80	53.16	0.070	-1.86	51.74	0.071
7.75	-3.84	53.57	0.072	-2.01	52.08	0.072
8.00	-3.86	54.01	0.074	-2.15	52.45	0.074
8.25	-3.88	54.47	0.075	-2.29	52.83	0.076
8.50	-3.89	54.96	0.077	-2.41	53.23	0.077
8.75	-3.89	55.46	0.079	-2.51	53.66	0.079
9.00	-3.88	55.96	0.080	-2.60	54.13	0.081
9.25	-3.87	56.45	0.082	-2.67	54.62	0.082
9.50	-3.85	56.94	0.084	-2.71	55.12	0.083
9.75	-3.81	57.40	0.085	-2.73	55.64	0.084
10.00	-3.78	57.86	0.087	-2.72	56.15	0.085
10.25	-3.74	58.31	0.089	-2.70	56.66	0.086
10.50	-3.71	58.77	0.090	-2.65	57.18	0.087
10.75	-3.67	59.23	0.092	-2.60	57.71	0.087
11.00	-3.63	59.70	0.093	-2.53	58.25	0.088
11.25	-3.60	60.18	0.095	-2.45	58.79	0.088
11.50	-3.56	60.68	0.097	-2.37	59.33	0.089
11.75	-3.52	61.18	0.098	-2.28	59.86	0.089
12.00	-3.48	61.67	0.100	-2.19	60.36	0.089
12.25	-3.44	62.17	0.102	-2.10	60.84	0.090
12.50	-3.39	62.67	0.103	-2.00	61.31	0.090
12.75	-3.35	63.16	0.105	-1.90	61.77	0.091

Additional file 2: Box–Cox values for power (L), adjusted mean (M), and coefficient of variation (S) of WC/Ht for the Japanese children of boys and girls from 4.50 to 12.75 years of age.

Age (years)	Boys			Girls		
	L	M	S	L	M	S
4.50	-2.62	0.46	0.049	0.82	0.47	0.046
4.75	-2.69	0.46	0.051	0.64	0.47	0.048
5.00	-2.76	0.46	0.053	0.45	0.46	0.050
5.25	-2.84	0.46	0.055	0.26	0.46	0.053
5.50	-2.91	0.46	0.057	0.07	0.46	0.055
5.75	-2.98	0.45	0.058	-0.12	0.45	0.057
6.00	-3.05	0.45	0.060	-0.31	0.45	0.059
6.25	-3.12	0.45	0.061	-0.5	0.45	0.061
6.50	-3.18	0.45	0.063	-0.68	0.44	0.063
6.75	-3.23	0.45	0.064	-0.87	0.44	0.064
7.00	-3.28	0.44	0.066	-1.07	0.44	0.066
7.25	-3.33	0.44	0.067	-1.26	0.43	0.067
7.50	-3.37	0.44	0.068	-1.45	0.43	0.069
7.75	-3.41	0.44	0.069	-1.64	0.43	0.070
8.00	-3.44	0.44	0.070	-1.82	0.43	0.071
8.25	-3.49	0.43	0.072	-1.99	0.42	0.073
8.50	-3.53	0.43	0.073	-2.15	0.42	0.074
8.75	-3.57	0.43	0.074	-2.29	0.42	0.075
9.00	-3.60	0.43	0.075	-2.41	0.42	0.076
9.25	-3.64	0.43	0.076	-2.52	0.42	0.076
9.50	-3.66	0.43	0.077	-2.61	0.42	0.077
9.75	-3.68	0.43	0.078	-2.69	0.41	0.077
10.00	-3.70	0.43	0.079	-2.77	0.41	0.078
10.25	-3.72	0.43	0.080	-2.83	0.41	0.077
10.50	-3.74	0.43	0.081	-2.90	0.41	0.077
10.75	-3.76	0.43	0.082	-2.96	0.41	0.077
11.00	-3.79	0.43	0.083	-3.02	0.41	0.076
11.25	-3.81	0.42	0.085	-3.09	0.41	0.076
11.50	-3.83	0.42	0.086	-3.15	0.41	0.075
11.75	-3.85	0.42	0.087	-3.21	0.41	0.075
12.00	-3.87	0.42	0.088	-3.26	0.41	0.074
12.25	-3.89	0.42	0.090	-3.31	0.40	0.074
12.50	-3.91	0.42	0.091	-3.35	0.40	0.074
12.75	-3.93	0.42	0.092	-3.40	0.40	0.074
